# Proteomic and Functional Analysis of the Effects of Quinoxaline Derivatives on *Entamoeba histolytica*

**DOI:** 10.3389/fcimb.2022.887647

**Published:** 2022-06-27

**Authors:** Rodolfo Gamaliel Avila-Bonilla, Ángel López-Sandoval, Jacqueline Soto-Sánchez, Laurence A. Marchat, Gildardo Rivera, Oscar Medina-Contreras, Esther Ramírez-Moreno

**Affiliations:** ^1^ Instituto Politécnico Nacional, Escuela Nacional de Medicina y Homeopatía, Laboratorio de Biomedicina Molecular 2, México City, Mexico; ^2^ Instituto Politécnico Nacional, Centro de Biotecnología Genómica, Laboratorio de Biotecnología Farmacéutica, Reynosa, Mexico; ^3^ Hospital Infantil de México Federico Gómez, Unidad de Investigación Epidemiológica en Endocrinología y Nutrición (UIEEN), México City, Mexico

**Keywords:** *E. histolytica*, quinoxaline derivatives, proteomics, functional analysis, Antiamoebic activity

## Abstract

Quinoxalines are heterocyclic compounds that contain a benzene ring and a pyrazine ring. The oxidation of both nitrogen of the pyrazine ring results in quinoxaline derivatives (QdNO), which exhibit a variety of biological properties, including antiparasitic activity. However, its activity against *Entamoeba histolytica*, the protozoan that causes human amebiasis, is poorly understood. Recently, our group reported that various QdNOs produce morphological changes in *E. histolytica* trophozoites, increase reactive oxygen species, and inhibit thioredoxin reductase activity. Notably, T-001 and T-017 derivatives were among the QdNOs with the best activity. In order to contribute to the characterization of the antiamebic effect of QdNOs, in this work we analyzed the proteomic profile of *E. histolytica* trophozoites treated with the QdNOs T-001 and T-017, and the results were correlated with functional assays. A total number of 163 deregulated proteins were found in trophozoites treated with T-001, and 131 in those treated with T-017. A set of 21 overexpressed and 24 under-expressed proteins was identified, which were mainly related to cytoskeleton and intracellular traffic, nucleic acid transcription, translation and binding, and redox homeostasis. Furthermore, T-001 and T-017 modified the virulence of trophozoites, since they altered their erythrophagocytosis, migration, adhesion and cytolytic capacity. Our results show that in addition to alter reactive oxygen species, and thioredoxin reductase activity, T-001 and T-017 affect essential functions related to the actin cytoskeleton, which eventually affects *E. histolytica* virulence and survival.

## Introduction

Quinoxalines are heterocyclic compounds made up of a benzene and pyrazine ring. The quinoxaline ring is described as a bioisostere of quinoline, naphthalene, benzothiophene and other aromatic cycles such as pyridine and pyrazine, some of which are the basis of known antimalarial and antitubercular agents for clinical use (Vicente et al., 2007). Quinoxalines 1,4-di-N-oxides are a class of quinoxaline derivatives that possess two N-O bonds at the N1 and N4 positions, respectively (Mu et al., 2014). The quinoxaline derivatives 1,4-di-N -oxides (QdNOs) are obtained by small modifications in the structure of the ring, especially in the positions R2, R3, R6 and R7, which have been related to differences in the biological activity and therefore selectivity (Leeson et al., 2004; Santivañez-Veliz et al., 2013). N-oxide groups are the main functional groups of quinoxaline derivatives (González et al., 2007). The presence of N-oxide groups in QdNOs allows their varied biological properties, including their effects against protozoa that affect human health, such as *Plasmodium falciparum* (Bonilla-Ramirez et al., 2018), *Trypanosoma cruzi* and *Leishmania mexicana* (Chacón-Vargas et al., 2017), and *Trichomonas vaginalis* (Carta et al., 2004). Although some mechanisms have been described in several protozoa, very little is known about the activity of QdNOs against *Entamoeba histolytica*, the protozoan that causes human amoebiasis. This disease is one of the main causes of mortality and morbidity in developing countries, being the third parasitic disease with the highest mortality after malaria and schistosomiasis (WHO, 1998). Although the drug of choice for the treatment of intestinal and extraintestinal amoebiasis is metronidazole, its use produces adverse effects and has been associated with carcinogenesis (Rustia and Shubik, 1972), mutagenesis and teratogenesis (Kazy et al., 2005). Additionally, several strains of *E. histolytica* resistant to metronidazole have been obtained in the laboratory (Upcroft and Upcroft, 1993; Upcroft and Upcroft, 2001), which evidences the necessity of developing new drugs to control this parasitosis.

Duque-Montaño et al. (2013) evaluated the effect of 25 new compounds derived from QdNOs which had various substituents at the R2, R3 and R7 positions, on cultures of *E. histolytica* trophozoites (HM1: IMSS strain). Of these 25 ethyl and methyl quinoxalines 7-carboxylate 1,4-di-N-oxide (7-carboxylate QdNOs), 10 showed a better antiamebic activity than metronidazole, the reference drug. Moreover, by comparing the antiamebic activity and cytotoxic activity in Vero cells, the compounds that presented the best selectivity index (SI) were T-017 (SI> 51.81), T-014 (SI> 53.19), T-001 (IS> 68.02) and T-016 (IS> 70.92), suggesting that these molecules could be valuable candidates as antiamebic drugs (Duque-Montaño et al., 2013). Recently, Soto-Sánchez et al., 2020, showed that these four 7-carboxylate QdNOs produce morphological changes in *E. histolytica* trophozoites, including chromatin remodelling, cellular granularity and redistribution of vacuoles, as well as an increase in reactive oxygen species (ROS). Additionally, molecular docking analysis indicated that the compounds can interact with amino acid residues of the NADH-binding domain and the redox active site of *E. histolytica* thioredoxin reductase (EhTrxR); complementary enzyme assays showed that the compounds inhibit its disulfide reductase and diaphorase activity, acting as electron acceptor substrate for this enzyme.

In this work, we selected T-001 and T-017 that have the best antiamebic properties and evaluate their effect on protein expression in *E. histolytica.* We also performed functional assays to assess the impact of deregulated proteins on parasite virulence properties that depends on cytosqueleton dynamics, which complements our knowledge about the mechanisms of action of QdNOs on this parasite.

## Materials and Methods

### Synthetic Compounds

Quinoxaline derivatives T-001 (Methyl 2-acetyl-3-methyl-quinoxaline-7-carboxylate 1,4-di-N-oxide) and T-017 (Ethyl 2-benzoyl-3-methylquinoxaline-7-carboxylate 1,4-di-N-oxide) were synthesized as described (Gómez-Caro et al., 2011). Stock solutions were prepared in DMSO (the concentration used in the experiments did not exceed 0.01%) and each compound was prepared at a final concentration of 1 mg/mL. Metronidazole (Sigma-Aldrich) was used as a reference drug.

### *E. histolytica* Culture and Treatments

*E. histolytica* trophozoites (HM1: IMSS strain) were axenically cultured in 75 cm^2^ culture flasks at 37°C in TYIS-33 medium supplemented with 16.8% (v/v) heat-inactivated adult bovine serum (Diamond et al., 1978), 3.2% (v/v) Diamond Vitamin-Tween 80 solution (Sigma-Aldrich), 100 U/mL penicillin, and 100 μg/mL streptomycin. Trophozoites were treated for 48 h at 37°C with the IC_50_ of T-001 (1.41 µM) or T-017 (1.93 µM), previously reported by Duque-Montaño et al. (2013). Parasites without treatment were included as controls. This experiment was carried out in triplicate.

### Protein Extraction and Cleaning

Trophozoites were centrifuged and washed with PBS pH 6.8 at 4°C and resuspended in 1 ml buffer lysis, (50 mM Tris pH 7.4, 0.25% SDS, protease inhibitors: 0.04 mM E-64, 100 µM Leupeptin, 7 mM PMSF, 8.75 mM iodoacetamide and protease inhibitor cocktail [Complete, Roche]). After homogenization, trophozoites were lysed by freeze-thaw cycles; the solution was centrifuged at 16,000 g for 5 min at 4°C and the supernatant was distributed in aliquots in Eppendorf tubes and stored at -80°C, until use.

Protein cleaning and purification were carried out by acetone precipitation, the proteins were obtained by centrifugation at 21,000 g for 5 min at 4°C, the pellet was allowed to air dry and resuspended in rehydration buffer (7 M urea, 2 M thiourea, 2% CHAPS, 40 mM DTT, 0.5% ampholytes 4-7, and traces of bromophenol blue).

### 2D-DIGE (Two-Dimensional-Differential-in-Gel-Electrophoresis) Analysis

Protein extract (500 µg) were applied to acrylamide gel strips with an immobilized linear gradient of pH 4.0-7.0 (ReadyStrip ™ IPG strips pH 4.0-7.0, BioRad). Isoelectric focusing was performed with the Protean IEF i12 ™ cell system (BioRad), following the recommendations of the commercial company, under the following conditions: step 1: 250 V for 20 min, step 2: 8000 V for 1h, step 3: 8000 V for 26 h, step 4: 1500 V sustained. For the second dimension, the IPG strips were placed directly on the 12% polyacrylamide gel, along with 7 µL of pre-stained molecular weight marker (Bio-Rad). Electrophoresis was carried out at 80 V for 15 min, and at 200 V until the end of the run. The gel was immersed for 1 h in fixing solution (50% methanol, 10% acetic acid), washed three times with Milli-Q water for 10 min and stained with Sypro^®^ Ruby Protein Gel Stain (Invitrogen ™, Molecular Probes) according to the supplier recommendations. Finally, the stained gel was visualized with UV light through the ChemiDocs XRS system (Bio-Rad Laboratories), images were acquired using the Image lab 5.2.1 software (Bio-Rad Laboratories) and spots were detected with the PDQuest 8.0.1 software (Bio-Rad Laboratories). The protein profiles of the biological triplicates for each condition were normalized using a synthetic image called MatchSet master. Subsequently, the intensity of the spots was normalized according to the total intensity of the valid spots, in order to minimize possible errors due to differences in the amount of protein and intensity of the staining. The Student´s t test was used to determine any significant differences in spot intensity (p<0.05).

The protein profiles corresponding to the treatment with T-001 and T-017 were compared with that of trophozoites without treatment. Only those spots that showed a Fold change> 2-fold between control and treatments were considered as differentially expressed proteins and selected for subsequent analysis by mass spectrometry (MS), according to Student´s t test (p<0.05).

### Mass Spectrometry

A total of 45 spots were selected and excised with the EXQuest spot cutter (Bio-Rad Laboratories). They were bleached using acetonitrile (C_2_H_3_N) and 50 mM ammonium bicarbonate (NH_4_CO_3_) in a 1:1 ratio for 15 min, and then washed with 50 µL acetonitrile for 5 min. All the liquid was removed, and samples were dried in a vacuum centrifuge. Proteins were reduced with 10 mM DTT and 50 mM ammonium bicarbonate for 45 min at 56°C and alkylated with 55 mM iodoacetamide for 30 min at room temperature. Later, they were washed with sodium bicarbonate and acetronitrile, and digested with 0.1 µg/µL trypsin in 50 mM ammonium bicarbonate, overnight at 37°C. The peptides were extracted with 0.1% trifluoroacetic acid and 25 µL of acetronitrile for 30 min, the solvent was removed by lyophilization and peptides were submitted to a MALDI-TOF/TOF analyzer (Ultraflex III, Bruker, Germany). Mass spectra were acquired in positive ion mode and automatically submitted to Mascot software v.2.1 (http://www.matrixscience.com) for protein identification against the NCBI database for non-redundant proteins (http://www.ncbi.nlm.nih.gov/). As defined by Mascot probability analysis, only significant scores greater than “identity” (95% level of confidence) were considered to assign protein identity. All positive protein identification scores were significant (p < 0.05).

The identity of the proteins was corroborated in the amoeba (https://amoebadb.org/amoeba/) and Uniprot (https://www.uniprot.org) databases. Subsequently, they were categorized and classified according with their function, using the DAVID Bioinformatics Resources 6.8 (https://david.ncifcrf.gov/) and the PANTHER classification system (Protein Annotation Through Evolutionary Relationship) (http://www.pantherdb.org/). The proteins with unknow function were searched in the Argot 2 program (http://www.medcomp.medicina.unipd.it/Argot2/index.php). Finally, information related to the involvement of these proteins in various biological processes in *E. histolytica* was documented.

The mass spectrometry proteomics data have been deposited to the ProteomeXchange Consortium *via* the PRIDE (Perez-Riverol et al., 2022) partner repository with the dataset identifier PXD032322.

### Real-Time qRT-PCR

Total RNA was obtained from trophozoites previously treated with the respective quinoxaline derivatives T-001 and T-017, using TRIzol reagent (Thermo Fisher Scientific). RNA from trophozoites growing in standard conditions was used as control. Following DNase 1 treatment, cDNA synthesis was carried out using 5 µg of RNA and the SuperScript III First-Strand kit (Invitrogen). Specific primers for Peroxiredoxin putative (Forward, 5′-agcatggtgtgaagcagataa-3′; Reverse, 5′-cctgcttcgacatttaacattcc-3′), Actin 2 putative (Forward, 5′-atgggagacgaagaagttcaag-3′; Reverse, 5′-ttgacccataccagccataac-3′), Tyrosine kinase (Forward, 5′-cagagctgatggtcctccaa-3′; Reverse, 5′-tgaccaaatgacccagctcc-3′), Thioredoxin putative (Forward, 5′-ttgtgaatggtgtaaggaaatgag-3′; Reverse, 5′-gcactggctattaaagaatccatga-3′), Adapter protein (Forward, 5′-agcctcvtcttgattttgctcca-3′; Reverse, 5′-acacattgagaagccaaactagg-3′) and Cysteinyl-tRNA synthetase (Forward, 5′-caaaagtcggtgtacgtgttga-3′; Reverse, 5′-ttggatctgaagcccaactct-3′) were used in qPCR assays. Primers to amplify the RNA polymerase II gene (Forward, 5′-gatccaacatatcctaaaacaaca-3′; Reverse, 5′-tcaattattttctgacccgtcttc-3′) were used as internal control (Penuliar et al., 2012). The reactions were carried out in Fast Optical 48-Well Reaction Plates using 500 ng of cDNA, 5 µM of primers and 5 µL of Sensi FAST SYBR Hi-ROX master mix (Bioline). To ensure the presence of specific products, the denaturation curve (melt curve) was carried out and the relative expression of the genes was calculated by the ΔΔCT method (Livak & Schmittgen, 2001). Three independent and triplicate experiments were done. Data were analyzed using the Tukey test with the Sigma Stat version 2 software.

### Cell Migration Assay

Trophozoites previously treated for 48 h with the IC_50_ of T-001 or T-017 (as mentioned above), were incubated in TYI-S-33 medium without serum for 3 h at 37°C (Serum starvation) (Franco et al., 1997). They were chilled on ice for 5 min, centrifuged at 320 x g for 5 min and washed three times in TBS-CaCl_2_ buffer (50 mM Tris, 150 mM NaCl and 1 mM CaCl_2_, pH 7.2). Then, 5x10^4^ trophozoites were placed in the upper part of a transwell chamber (8 µm, Corning), while 600 µL of TYI-S-33 medium with serum was placed in the lower compartment. Cells were incubated at 37°C for 3 h and the number of trophozoites that migrated to the lower compartment was determined by counting in a Neubauer chamber and staining with 0.4% trypan blue (Gilchrist et al., 2008). The tests were carried out three times in triplicate. Untreated trophozoites and parasites treated with DMSO 0.01% were included as controls. The results were expressed as the mean of migration percentage ± standard deviation (SD). The comparisons between groups were carried out with the one way ANOVA test.

### Adhesion Assay

Trophozoites previously treated with the selected QdNOs were washed and resuspended in 1 mL of TYI-S-33 medium without serum; later they were seeded on monolayers of SW-480 cells previously cultured in 24-well plates, in a ratio of 1 trophozoite per 4 cells. The plates were incubated at 37°C for 15 min, after which the unattached trophozoites were removed from the culture medium, centrifuged at 320 x g for 5 min and counted using a Neubauer chamber. The tests were carried out in triplicate (Kobiler and Mirelman, 1981). Untreated trophozoites and parasites treated with DMSO 0.01% were included as controls. The results were expressed as the mean of adhesion percentage ± standard deviation (SD). The comparisons between groups were carried out with the one way ANOVA test.

### Cytolytic Effect

SW-480 cells were seeded in a 24-well plate (1.8x10^5^ cells/well) in supplemented RPMI-1640 medium and incubated 48 h at 37°C with 5% CO_2_. The cell monolayers were washed three times with PBS pH 6.8 and incubated at 37°C for 45 min with trophozoites (previously treated with selected QdNOs, untreated or DMSO treated, and resuspended in TYI-S-33 medium without serum) in 1:10 ration (amoeba: SW-480 cells). After incubation, the supernatant was removed, and lactate dehydrogenase activity was determined using the CytoTox 96^®^ Non-Radioactive Cytotoxicity Assay Kit, following the manufacturer instructions. Plates were read at 492 nm using a Multiskan FC reader (Thermo Scientific). SW-480 cells or trophozoites only with TYI-S-33 culture medium were used as negative controls; as lysis control, SW-480 cells lysed for 45 min with 9% X-100 triton. All conditions were tested in triplicate. Each experiment was repeated three times. The comparisons between groups were carried out with the one way ANOVA test.

### Erythrophagocytosis Assays

Human erythrocytes (0.5 x 10^8^ cells/mL) were mixed with an equal volume of *E. histolytica* trophozoites (0.5x10^6^ cells/ml). Both cell suspensions were prepared with serum-free medium. Cell mixture was incubated for 0, 5, 10 or 15 min at 37°C, subsequently fixed with 4% paraformaldehyde (Trissl et al., 1978), and washed three times with PBS 6.8 to remove non-phagocytosed erythrocytes. Ingested erythrocytes were contrasted by the Novikoff staining method (Novikoff et al., 1972) using fresh diaminobenzidine solution (3,3'-diaminobenzidine 2 mg/mL, 0.048% H_2_O_2_ and 50 Mm Tris-HCL, pH 9.7). The number of ingested erythrocytes was quantified and documented under an optical microscope. The results were expressed as the mean of ingested erythrocyte ratio ± standard deviation (SD). The comparisons between groups were carried out with the one way ANOVA test.

### Hemoglobin Quantification

Erythrophagocytosis capacity was evaluated by quantitative determination of hemoglobin within trophozoites. After 10 min of interaction of the amoeba trophozoites with human erythrocytes as described above, the trophozoites were recovered by washing three times with 1 mL of cold distilled water; then they were lysed with 1 mL formic acid and the amount of hemoglobin was measured by spectrophotometric analysis at 400 nm, using formic acid as blank (Voigt et al., 1999). The hemoglobin quantitation was obtained in relation to the untreated parasites. The results were expressed as the mean of hemoglobin ± standard deviation (SD). The comparisons between groups were carried out with the one way ANOVA test.

## Results

**Figure 1:**
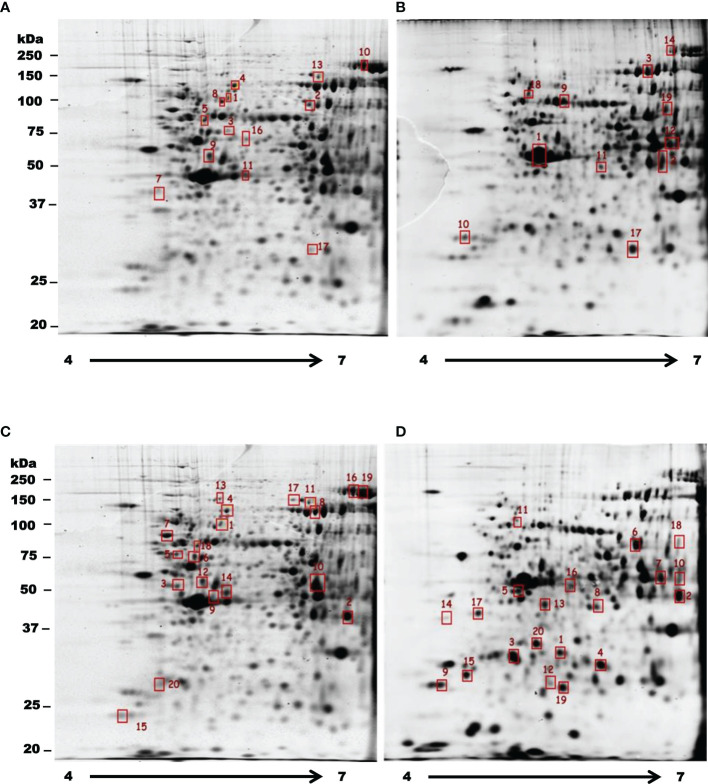
Two dimensional differential in gel electrophoresis of *E. histolytica* trophozoite proteins under the effect of T-001 and T-017. Selected spots for protein identification corresponding to proteins with a differential abundance between control trophozoites **(A, C)** and parasites treated with T-001 **(B)** or T-017 **(D)** are indicated in red squares. Underexpressed proteins are marked on gels of untreated trophozoites **(A, C)** and the overexpressed ones are indicated on gels of trophozoites treated with T-001 and T-017 **(B, D)**. The isoelectric focusing was carried out in a non-linear gradient of pH 4-7.

**Figure 2:**
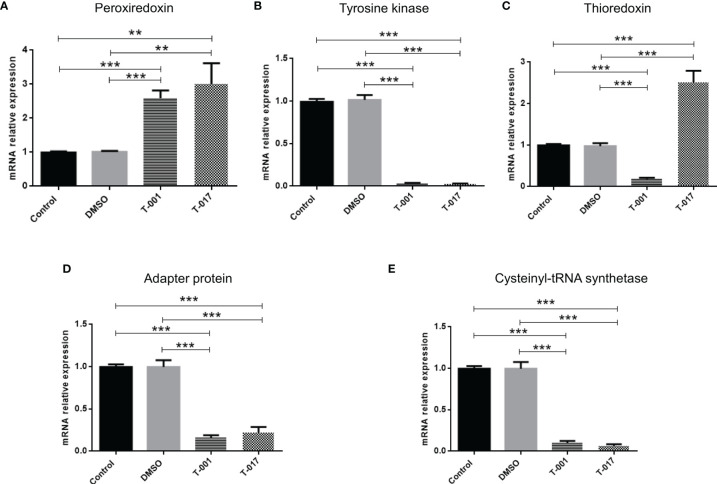
mRNA expression in *E. histolytica* trophozoites under the effect of T-001 or T-017. Trophozoites were treated with T-001 or T-017 during 48 h. Total RNAs were obtained and qRT-PCR was performed using Sensi FAST SYBR Hi-ROX master mix (Bioline), and specific primers to amplifying **(A)** Peroxiredoxin, **(B)** Tyrosine kinase, **(C)** Thioredoxin, **(D)** Adapter protein and **(E)** Cysteinyl-tRNA synthetase, as well as RNA Pol lI as endogenous control gene. Relative fold increase in gene expression was obtained using the ΔΔCT method. Data corresponds to mean ± SD, of three independent experiments. Statistically significant differences in mRNA expression were analyzed using a Tukey test method with Sigma Stat statistical software ver.2.0. p ≤ 0.01 (**), p ≤ 0.001 (***).

In order to observe how treatments with T-001 and T-017 affect protein expression, trophozoite extracts were analyzed by two-dimensional electrophoresis, and proteins were visualized by Sypro Ruby staining. The gels corresponding to treated and untreated trophozoites showed an efficient protein separation (**Figure 1**). Many of the detected amoebic proteins have an isoelectric point (IP) of 5 to 6 and a molecular weight between 20-100 kDa. A total of 669 spots were visualized in untreated trophozoites, whereas 590 and 657 spots were found in proteins extracts from T-001 and T-017 treated cells, respectively. Analysis with the 2D-PD QUEST program revealed that T-001 modified the expression of 163 proteins, 79 were overexpressed and 84 were under expressed compared to the untreated group. Treatment with T-017 produced 131 differentially expressed spots, 70 overexpressed and 61 under expressed (>2 fold change). A set of 24 spots modulated by T-001 (11 overexpressed and 13 under-expressed) (**Table 1**), and 21 spots modulated by T-017 (10 overexpressed and 11 under-expressed) (**Table 2**) were selected for protein identification by mass spectrometry. The proteins with the highest fold change after T-001 treatment were the truncated hsp 70 family protein that was overexpressed with a fold change of 7.26, while the Fbox/WD domain containing protein and the tyrosine kinase were underexpressed with a fold change of 7.87 and 6.08, respectively. On the other hand, the proteins modulated by T-017 with the highest fold change were the Rab GTPase protein, overexpressed with a fold change of 6.42, while the adenylylcyclase associated protein was underexpressed with a fold change of 6.06. Changes in protein abundance were corroborated by Real-time RT-qPCR assays to evaluate the relative mRNA expression of six proteins that were similarly deregulated by T-001 and T-017 (**Figure 2**). Interestingly, the overexpression of the putative peroxyredoxin gene, and the under-expression of tyrosine kinase, thioredoxin, adapter protein (AP) and cysteinyl-tRNA synthetase genes agreed with the results of protein expression.

**Table 1:** Identification of *E. histolytica* proteins that are modulated by QdNO T-001.

Protein(Spot number)	Accession number	Fold change	Mascot score	Sequence coverage %	Molecular weight (kDa)/IP
**Overexpressed**					
Actin 2 protein, putative (1)	B1N5D1	4.45	34	65	81000/5.51
Guanine nucleotide-binding protein subunit beta 2-like 1, putative (2)	C4M6P6	2.01	34	22	35077/6.82
Truncated hsp70 family protein (3)	C4MBG4	7.26	38	53	13018/9.55
Vesicle-fusing ATPase (9)	C4LYS4	4.06	38	18	82693/6.05
40S ribosomal protein S24, putative(10)	C4M549	2.23	48	37	16260/10.39
TBC domain containing protein (11)	C4M7P5	2.44	38	28	36.208/6.19
Hypothetical protein (12)	C4M893	2.97	30	27	21.521/9.57
Protein phosphatase, putative (14)	C4LU84	3.01	25	23	36607/4.59
Peroxiredoxin, putative (17)	B1N5Y9	2.07	34	69	14744/7.61
Hypothetical protein (18)	B1N2E4	2.36	54	29	28062/8.22
Calmodulin, putative(19)	C4LTA2	2.69	20	96	6265/12.03
**Underexpressed**					
Hypothetical protein (1)	C4M061	4	39	31	40357/6.2
F-box/WD domain containing protein (2)	C4LUA3	7.87	39	18	84985/7.98
Hypothetical protein (3)	C4LZE3	2.45	29	15	56661/6.99
Pyruvate, phosphate dikinase (4)	Q24801	2.72	53	39	97899/5.89
Cysteinyl-tRNA synthetase, putative (5)	C4M8V1	2.34	40	16	80198/7.24
Hypothetical protein (7)	C4LWP4	2.78	30	22	22832/6.7
Hypothetical protein (8)	C4LZ44	2.32	29	14	35973/5.95
Hypothetical protein (9)	C4M9A0	2.8	34	45	13421/6.05
Tyrosine kinase, putative (10)	C4M315	6.08	34	17	125598/4.77
PCI domain containing protein (11)	C4M536	3.73	30	19	36088/6.23
Adapter protein (AP) family protein (13)	C4M5F2	2.81	37	7	98994/7.12
Hypothetical protein (16)	C4M4Z1	2.07	29	24	27087/8.6
Fumarate hydratase class I, anaerobic, putative (17)	C4MB76	2.51	42	32	22311/7.09

IP (isoelectric point) and molecular weight were calculated using the EXPASY software (https://web.expasy.org/compute_pi/).

**Table 2:** Identification of *E. histolytica* proteins that are modulated by QdNO T-017.

Protein (Spot number)	Accession number	Fold change	Mascot score	Sequence coverage %	Molecular weight (kDa)/IP
**Overexpressed**					
Rab GTPase (1)	Q5NT06	6.42	32	19	21448/5.32
60S ribosomal protein L27, putative (3)	C4M727	2.48	39	42	15758/10.44
Thioredoxin, putative (4)	C4LSU6	2.86	45	54	14811/6.58
Ribosome biogenesis protein, putative (5)	C4LVY6	5.48	32	17	36895/9.75
Hypothetical protein (9)	C4M091	2.87	40	61	13913/7.61
Hypothetical protein (13)	C4M630	1.86	40	39	36797/9.43
Hypothetical protein (15)	B1N2U7	4.36	40	52	11306/5.05
AIG1 family protein (17)	B1N4J0	3.25	48	12	35560/5.09
ADP-ribosylation factor 1, putative (19)	Q1EQ60	2.79	36	37	19512/5.29
Grainin, putative (20)	B1N4A1	2.47	40	52	24599/6.73
**Underexpressed**					
Hypothetical protein (1)	C4M061	2.28	39	31	40357/6.2
Hypothetical protein (3)	C4M3S7	2.14	40	9	75471/6.78
Pyruvate phosphate dikinase (4)	Q24801	2.77	53	39	97899/5.89
Adenylylcyclase-associated protein, putative (5)	B1N3T9	6.06	31	28	18449/5.05
F-box/WD domain containing protein (6)	C4LUA3	3.63	39	18	84985/7.98
Hypothetical protein (7)	C4M9A0	3.51	34	45	13421/6.05
Adapter protein (AP) family protein (11)	C4M5F2	3.44	37	7	98994/7.12
Hypothetical protein (12)	C4M3R3	2.22	34	17	62.140/8.8
Importin beta-3 family protein (13)	C4LXC2	2.95	30	6	125550/5.1
Hypothetical protein (17)	C4M563	3.68	42	19	123240/8.51
Cysteinyl-tRNA synthetase, putative (18)	C4M8V1	2.10	59	18	80198/7.24

IP (isoelectric point) and molecular weight were calculated using the EXPASY software (https://web.expasy.org/compute_pi/).

### Treatments With QdNOs T-001 and T-017 Modulate Proteins Involved in Various Biological Processes in Trophozoites of *E. histolytica*

To categorize the function of the modulated proteins, the DAVID Bioinformatics Resources 6.8 bionformatic program (https://david.ncifcrf.gov/) and the PantherDB database (http://www.pantherdb.org/) were used. Those proteins whose function was not found were searched in the Argot 2 program (Functional annotation of proteins using the semantic similarity in the Gene Ontology). **Table 3** shows the function of proteins deregulated by compound T-001. Interestingly, these include proteins related with translation (guanine nucleotide-binding protein subunit beta 2-like 1, 40S ribosomal protein S24 and cysteinyl-tRNA synthetase), intracellular traffic (TBC domain containing protein, vesicle-fusing ATPase and adapter protein), phosphorylation (hypothetical protein [3] and [16], and tyrosine kinase), cytoskeletal organization (actin 2 and calmodulin), energy generation (pyruvate phosphate dikinase and fumarate hydratase class I), nucleic acid binding (hypothetical protein [8] and PCI domain containing protein), redox homeostasis (peroxiredoxin), stress response (truncated hsp70 family protein), ubiquitination (hypothetical protein [12]), kinase activity (hypothetical protein [1]), transport (hypothetical protein [7]) and proteins with unknown function (hypothetical proteins [9] and [18], and F-box/WD domain containing protein). On the other hand, proteins deregulated by compound T-017 were related with intracellular traffic (Rab GTPase family protein, ADP-ribosylation factor 1, importin beta-3 family protein and adapter protein), nucleic acid binding (hypothetical protein [9] and [1]), translation (60S ribosomal protein L27 and cysteinyl-tRNA synthetase), binding to calcium ion (grainin), hydrolase activity (hypothetical protein [15]), redox homeostasis (thioredoxin), rRNA processing (ribosome biogenesis protein), stress response (AIG1 family protein), energy generation (pyruvate phosphate dikinase), post-traslational modifications (hypothetical protein [17]), cytoskeletal organization (adenylylcyclase-associated protein), post-transcriptional processing (hypothetical protein [12]) and proteins with unknown function (hypothetical protein [3], [7] and [13], and F-box/WD domain containing protein) (**Table 4**).

**Table 3:** Functional categorization of *E. histolytica* proteins modulated by treatment with T-001.

Protein (Spot number)	Amoeba BD number	Function	Localization
**Overexpressed**			
Actin 2 protein, putative (1)	EHI_161200	Cytoskeletal organization	Actin Cytoskeleton
Calmodulin, putative(19)	EHI_044820	Cytoskeletal organization	Cytoplasm
TBC domain containing protein	EHI_091080	Intracellular traffic	Membrane
Vesicle-fusing ATPase (9)	EHI_004640	Intracellular traffic	Cytoplasm
Guanine nucleotide-binding protein subunit beta 2-like 1, putative (2)	EHI_171280	Traslation	Cytoplasm, nucleus
40S ribosomal protein S24, putative(10)	EHI_148820	Traslation	Cytoplasm
Protein phosphatase, putative (14)	EHI_110320	Phosphatase activity	Membrane
Hypothetical protein (18)	EHI_153630	Unknown function	Membrane
Peroxiredoxin, putative (17)	EHI_121620	Cell redox homeostasis	Cytoplasm
Truncated hsp70 family protein (3)	EHI_147220	Stress response	Cytoplasm
Hypothetical protein (12)	EHI_076020	Protein ubiquitination	Membrane
**Underexpressed**			
Hypothetical protein (3)	EHI_137800	Protein phosphorylation	Membrane
Tyrosine kinase, putative (10)	EHI_025280	Protein phosphorylation	Membrane
Hypothetical protein (16)	EHI_035240	Protein phosphorylation	Cytoplasm
F-box/WD domain containing protein (2)	EHI_110540	Unknown function	Cytoplasm
Hypothetical protein (9)	EHI_074500	Unknown function	Membrane
Hypothetical protein (8)	EHI_009700	Nucleic acid binding	No Found
PCI domain containing protein (11)	EHI_078210	Nucleic acid binding	Cytoplasm, nucleus
Pyruvate, phosphate dikinase (4)	EHI_009530	Energy generation (glycolysis)	Cytoplasm
Hypothetical protein (1)	EHI_021470	Kinase activity	Membrane
Cysteinyl-tRNA synthetase, putative (5)	EHI_169700	Cysteinyl-tRNA aminoacylation (translation)	Cytoplasm
Fumarate hydratase class I, anaerobic, putative (17)	EHI_117270	Generation of precursor metabolites and energy	Cytoplasm
Adapter protein (AP) family protein (13)	EHI_058450	Intracellular traffic	Cytoplasm
Hypothetical protein (7)	EHI_069560	Transport	Membrane

**Table 4:** Functional categorization of *E. histolytica* proteins modulated by treatment with T-017.

Protein (Spot number)	Amoeba BD number	Function	Localization
**Overexpressed**			
Rab GTPase family (1)	EHI_096440	Intracellular traffic	Cell
ADP-ribosylation factor 1, putative (19)	EHI_121870	Intracellular traffic	Cytoplasm
Grainin, putative (20)	EHI_120360	Binding to calcium ions	Cytoplasm
Hypothetical protein (9)	EHI_004970	Nucleic acid binding	No found
Hypothetical protein (15)	EHI_159530	Hydrolase activity	Membrane
Hypothetical protein (13)	EHI_125400	Unknown function	Membrane
Thioredoxin, putative (4)	EHI_152600	Redox homeostasis	Cell
Ribosome biogenesis protein, putative (5)	EHI_126010	rRNA processing	Nucleus
AIG1 family protein (17)	EHI_067730	Stress response	Membrane
60S ribosomal protein L27, putative (3)	EHI_183480	Translation	Cell component (ribosomal subunit)
**Underexpressed**			
Importin beta-3 family protein (13)	EHI_098400	Intracellular traffic	Cytoplasm; nucleus
Adapter protein (AP) family protein (11)	EHI_058450	Intracellular traffic	Cytoplasm
Hypothetical protein (7)	EHI_074500	Unknown function	Membrane
Hypothetical protein (3)	EHI_052890	Unknown function	Membrane
F-box/WD domain containing protein (6)	EHI_110540	Unknown function	Membrane
Pyruvate phosphate dikinase (4)	EHI_009530	Catalytic activity	No found
Cysteinyl-tRNA synthetase, putative (18)	EHI_169700	Cysteinyl-tRNA aminoacylation	Cytoplasm
Hypothetical protein (17)	EHI_178100	Post-translational modification	Membrane
Adenylylcyclase-associated protein, putative (5)	EHI_081430	Cytoskeletal organization	Actin cytoskeleton
Hypothetical protein (12)	EHI_146210	post-transcriptional processing	No found
Hypothetical protein (1)	EHI_021470	Nucleic acid binding	Membrane

Interestingly, both quinoxaline derivatives affect proteins that participate in the dynamics of the actin cytoskeleton and intracellular trafficking. Notably, Actin 2 and calmodulin overexpression was evidenced in trophozoites treated with T-001, while the protein associated with adenylyl cyclase were found to be downregulated by T-017. On the other hand, proteins associated with trafficking as TBC domain containing protein, vesicle-fusing ATPase and adapter protein were deregulated by compound T-001, while T-017 deregulated Rab GTPase family protein, ADP-ribosylation factor 1, importin beta-3 family protein and adapter protein. The cytoskeleton is particularly relevant for the pathogenicity mechanisms of amoeba. Adhesion is one of the first steps in the pathogenesis of *E. histolytica* and a prerequisite for host cell destruction. Together with the motility and ability to engulf host cells, adhesion allows trophozoites to colonize, invade and destroy different human tissues. Therefore, we decided to evaluate the impact of T-001 and T-017 treatment on migration, phagocytosis, and adhesion in *E. histolytica*; changes in the cytopathic effect of this parasite were also evaluated.

### T-001 and T-017 Decrease Migration, Adhesion and the Cytolytic Effect of *E. histolytica* Trophozoites

**Figure 3:**
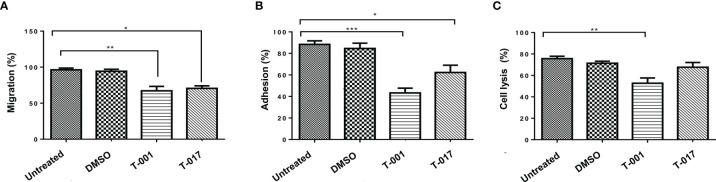
Effect of T-001 and T-017 on migration **(A)**, adhesion **(B)** and cytolytic activity **(C)** of (*E*) *histolytica* trophozoites. Trophozoites were previously treated with T-001 or T-017 as described. **(A)** Trophozoite migration was determined after 3 h incubation in transwell chambers and cell counting; migration was expressed as percentage. **(B)** Trophozoites were seeded on SW-480 epithelial cell monolayer during 15 min, adhesion were determined and expressed as percentage. **(C)** SW-480 cell monolayers were incubated with *E. histolytica* trophozoites for 45 min; damage to the SW-480 cell monolayer was evaluated by the release of the LDH enzyme to the culture medium and (absorbance at 492nm) expressed as percentage; the histogram shows the average percentage of destruction of the SW-480 monolayers. Trophozoites treated with DMSO 0.01% and grown in standard condition were used as controls. Graphs show the mean ± standard error of three independent experiments. The comparisons between groups were carried out with the one way ANOVA test, p < 0.05 (*), p < 0.01 (**), p < 0.001 (***).

To evaluate the effect of quinoxaline derivatives on the migration of amoeba trophozoites, an assay was carried out using transwell chambers. The trophozoites treated with T-001 showed a decrease of 27.14% in their migration with respect to untreated amoebae; similarly, compound T-017 produced a 23.77% decrease in the migration of trophozoites (**Figure 3A**). Likewise, to evaluate if quinoxaline derivatives could affect the adhesion of ameba trophozoites, an assay was carried out to evaluate trophozoite adhesion to SW-480 cell cultures. As shown in **Figure 3B**, both compounds caused a reduction in trophozoites adhesion, being compound T-001 the one that produced the greatest effect with a reduction of 49% compared to untreated trophozoites, while compound T-017 reduced adhesion in 27%. To evidence whether the decrease in adhesion caused by T-001 and T-017 compounds could affect the cytolytic activity of the amoeba trophozoites, we carried out interaction tests between SW-480 cell cultures and trophozoites at 37°C. After 45 min of interaction with untreated trophozoites, cell cultures showed an average of 75.66% destruction (**Figure 3C**). Similarly, amoebae treated with 0.01% DMSO showed 71.33% cell destruction. On the contrary, trophozoites treated with T-001 caused a lower degree of damage, since they only produced 52.66% of destruction. However, T-017 treatment did not significantly affect the ability of trophozoites to destroy SW-480 cell monolayers, since trophozoites treated with T-017 produced 67.66% of cell destruction.

### T-001 and T-017 Affect Erythrophagocytosis of *E. histolytica*

**Figure 4:**
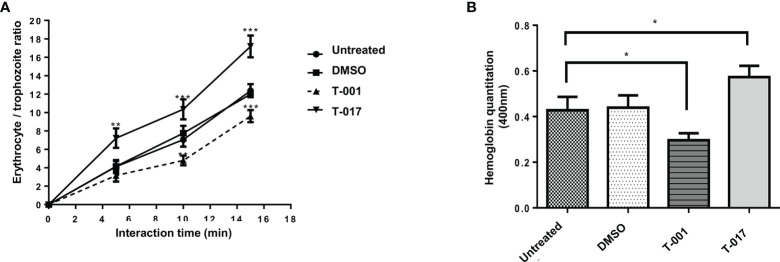
Erythrophagocytosis of *E. histolytica* trophozoites treated with T-001 and T-017. **(A)** Erythrophagocytosis rate at 0, 5, 10 and 15 min of interaction of erythrocytes and trophozoites previously treated with T-001 and T-017. **(B)** Hemoglobin quantitation after 10 min interaction of erythrocytes and trophozoites previously treated with T-001 and T-017 and untreated. Trophozoites treated with DMSO 0.01% and grown in standard condition were used as controls. Graphs show the mean ± standard error of three independent experiments. The comparisons between groups were carried out with the one way ANOVA test, p < 0.05 (*) p < 0.01 (**) p < 0.001 (***).

The effect of quinoxaline derivatives on the phagocytic capacity of amoeba trophozoites was carried out by interacting amoeba trophozoites with human erythrocytes. For this analysis, the number of erythrocytes ingested by amoeba trophozoites (previously treated with T-001 and T-017) was determined after 0, 5, 10 and 15 min of interaction. The results were visualized through staining with diaminobenzidine. As shown in **Figure 4**, this is a time-dependent process in untreated trophozoites since there is a progressive increase in the number of ingested erythrocytes through time. Trophozoites treated with 0.01% DMSO presented no significant difference with respect to untreated trophozoites. Interestingly, in the case of T-001 treatment, the erythrophagocytosis rate was significantly decreased by 32% and 23% at 10 and 15 minutes of interaction, respectively in reference to untreated group. In contrast, T-017 treatment increased erythrophagocytosis by about 50% and 42% at the same interaction times, with respect to untreated trophozoites. (**Figure 4A**). These results were corroborated by measuring the hemoglobin contained in the trophozoites after ten minutes of interaction with red blood cells; as shown in **Figure 4B**, the amount of hemoglobin was decreased by 30% and increased by 32%, in trophozoites treated with T-001 and T-017, respectively (**Figure 4B**).

## Discussion

QdNOs are a group of heterocyclic compounds characterized by a broad spectrum of biological activity. They are used as therapeutic agents in the treatment of various infections (Ishikawa et al., 2012; Lee et al., 2013; Kaplum et al., 2016) and inhibit the growth of protozoan parasites such as *P. falciparum* (IC50 = 1.25 µM) (Aldana et al., 2003), *P. falciparum* FCR-3 sensitive to chloroquine (IC50 = 0.40 µM) (Bonilla-Ramirez et al., 2018), *L. amazonensis* (IC50 = 0.74 µM) (Chacón-Vargas et al., 2018), *T. cruzi* (IC50 = 2.42 µM) (Chacón-Vargas et al., 2017), *T. vaginalis* (MIC = 0.39 µg/mL) (Carta et al., 2004) and *E. histolytica* (Soto-Sánchez et al., 2020). In order to contribute to the characterization of the antiamebic effect of two QdNOs with the highest inhibitory effect, in this work we analyzed the proteomic profile of *E. histolytica* trophozoites treated with T-001 and T-017 molecules and assessed the impact of deregulated proteins by functional assays.

The T-001 and T-017 QdNOs affect the abundance of 163 and 131 proteins, respectively. Among them, mass spectrometry assays allowed the identification of 24 proteins deregulated by T-001 (11 were found to be overexpressed and 13 under-expressed), while 10 over-expressed and 11 under expressed proteins were identified in T-017 treated parasites. The relative mRNA expression levels of genes corresponding to Peroxiredoxin, Tyrosine kinase, putative Thioredoxin, Adapter protein and Cysteinyl-tRNA synthetase agreed with the differential amount of these proteins in the presence of QdNOs, which validate the proteomic data. Although study of mRNA expression is not the best tool to validate protein expression, it is useful in microorganisms, such as *E. histolytica*, where there are no commercial antibodies to perform western blot assays.

Modulated proteins are associated with various cellular processes, such as intracellular trafficking, organization of the cytoskeleton and redox homeostasis, among others. Intracellular trafficking, including endocytosis and exocytosis, allows the transport of proteins and other macromolecules through organelles of the endomembrane system (Herman et al., 2017). Membrane trafficking interconnects the nuclear envelope, endoplasmic reticulum (ER), golgi apparatus, and various secretory vesicles. *E. histolytica* lacks visible classical organelles such as mitochondria and ER, although it has functions and proteins associated with Golgi/ER (Dacks et al., 2003; Bredeston et al., 2005; Teixeira and Huston, 2008). Additionally, the presence of continuous ER compartments has been suggested (Teixeira and Huston, 2008). Other studies support the existence of components of the trans Golgi network similar to those in mammals, but with a peculiar compartmentalization, also suggesting a very active membrane trafficking process (Perdomo et al., 2015). In trophozoites treated with quinoxalines T-001 and T-017, the expression of the adapter protein AP (C4M4Z1), which belongs to the family of AP-1, AP-2 and AP-3 involved in endosomal trafficking and endocytosis on the cell surface, was found to be decreased. Likewise, compound T-017 produced a decrease in the expression of putative beta 3 importin whose function has not yet been characterized in amoeba, however it is known that in other organisms this protein participates in the nuclear import of proteins that carry nuclear localization signals, through interactions with importin α, thus forming a trimeric complex (Gilchrist et al., 2002). Interestingly, the quinoxalines T-001 or T-017 also produced the overexpression of proteins of intracellular traffic: the protein with TBC domain (C4M7P5) and the protein of the Rab GTPase family (Q5NT06), both involved in the dynamics of the vesicular fusion (some Rabs contain the TBC domain), the ADP-ribosylation factor (ARFs) (Q1EQ60), a regulator of vesicle formation in intracellular traffic (Serbzhinskiy et al., 2015) and the putative vesicle-fusion ATPase (C4LYS4), which catalyzes the fusion of transport vesicles within the cisternae of the Golgi apparatus (Qiu, 2012). The deregulation of these proteins that participate in the intracellular traffic could explain the presence of a higher number of vacuoles and vesicles that we previously reported in *E. histolytica* trophozoites treated with T-001 and T-017 (Soto-Sánchez et al, 2020). Other studies carried out in *T. cruzi* epimastigotes also evidenced that QdNOs produced an increase in the number of vesicles in the cytoplasm was also found, as well as alterations in the Golgi apparatus, which suggest that QdNOs alters the secretory pathway in this parasite (Rodrigues et al., 2014).

On the other hand, it has been documented that under conditions of oxidative stress, such as that produced by quinoxalines, there is an active commitment of the *E. histolytica* cytoskeleton to provide support for survival functions, that is, migration, adhesion, phagocytosis, etc. (Pineda and Perdomo, 2017). Our results coincide with this observation since trophozoites treated with quinoxalines had an increase in the expression of actin 2 (B1N5D1), putative calmodulin (C4LTA2) and the adenyl cyclase-associated protein (B1N3T9). The actin protein is essential for the functions of the cytoskeleton, being necessary for the modulation between its filamentous (F-actin) and globular (G-actin) forms (Manich et al., 2018). One of the proteins that control actin dynamics is adenylate cyclase (AC) (Zhang et al., 2013), whose G-protein-mediated activation leads to changes in actin structure and effect on adhesion and movement of trophozoites (Soid-Raggi et al., 1998). Regarding the calmodulin-like protein, even though this typical protein has not been seen in *E. histolytica*, it has a large number of Ca^2+^ binding proteins, some of which can bind directly to actin and modulate its dynamics, a phenomenon that has not been seen in any other system (Babuta et al., 2020). It is likely that the overexpression of these proteins explains the alterations observed in the functionality of the cytoskeleton in trophozoites treated with T-001 and T-017, namely a decreased migration, adhesion and cytolysis, as well as alterations in erythrophagocytosis.

Previous works reported that quinoxaline derivatives inhibit enzymes that counteract oxidative stress, such as trypanothion reductase in *Trypanosoma cruzi* (Chacón-Vargas et al., 2017) and peroxiredoxin in *Toxoplasma gondii* (Haraldsen et al., 2009).

Quinoxaline-induced oxidative stress also affects enzyme systems in amoeba. In a previous study, we demonstrated that QdNOs T-001 and T-017 induced oxidative stress and inhibition of amoeba thioredoxin reductase (EhTrxR) activity (Soto-Sánchez et al., 2020). In the present work, we found that the expression of thioredoxin (C4LSU6) is upregulated in trophozoites treated with T-001; this supports the effect of quinoxalines on the thioredoxin-thioredoxin reductase system, which participates in the amoeba antioxidant defense, protecting sensitive proteins against oxidative stress (Schlosser et al., 2013). In this system, electrons are transferred from NADPH *via* FAD to the active disulfide site of TrxR, which reduces thioredoxin. Reduced thioredoxin interacts with target proteins involved in various biological processes, such as the degradation of reactive oxygen species (Andrade and Reed, 2015). Additionally, peroxiredoxin, an antioxidant protein that interacts with thioredoxin in its reduced state, was also found to be more abundant in trophozoites treated with T-017. Peroxiredoxin reduces peroxides, such as H_2_O_2_, hydroperoxides, and peroxynitrites, using thioredoxin and its dithiol motif as the hydrogen donor, thereby establishing the enzymatic redox cascade that mediates the flow of electrons from NADPH to TrxR and then to the thioredoxin. The reduced form of thioredoxin is then able to interact with peroxiredoxin (Gwairgi and Ghildyal, 2018). *In vitro*, an increase in the levels of the peroxiredoxin gene of *E. histolytica* HM1: IMSS has been seen in response to an environment with high oxygen content (Akbar et al., 2004). It is also known that *E. histolytica* moderately increases peroxiredoxin mRNA levels when it is incubated with 50 μM of metronidazole which, during its reoxidation, generates reactive oxygen species (Tazreiter et al., 2008).

Even though both compounds show antiamoebic activity, modulating proteins that participate in common events and impacting on similar pathogenicity mechanisms, there are clear differences in the modulated proteins, as well as in the degree of impact they have on the parasite. Compounds T-001 and T-017 differ in having a methyl group or a benzyl group in the R2 position of the quinoxaline ring, respectively, which suggests that these compounds may be interacting differently with their potential molecular targets, producing variations in their biological effect. In a previous study using molecular docking, we evaluated the interaction of T-001 and T-017 with thioredoxin reductase from *E. histolytica*, finding that structure affects binding energy and interactions with amino acids of the redox active site and/or the NADPH binding site; In addition, it was also found that quinoxalines with different substituents in the R2 position and with an increase in the aliphatic chain in the R3 position of the quinoxaline ring produced different changes in the morphology and ultrastructure of the amoeba trophozoite (Soto-Sánchez et al., 2020). Thus, the structural differences between T-001 and T-017 may explain the differences in their antiamoebic effect.

Altogether, our results indicate that the quinoxaline derivatives T-001 and T-017 exert their antiamebic activity by modulating the expression of proteins related with various mechanisms, such as intracellular traffic, organization of the cytoskeleton and redox homeostasis, among others, which impact on basic trophozoite functions, including migration, adhesion, cytolysis and phagocytic capacity, that eventually leading to the death of the parasite.

## Data Availability Statement

The datasets presented in this study can be found in online repositories. The name of the repository and accession number can be found below: PRIDE, ProteomeXchange; PXD032322.

## Author Contributions

RA-B: Real-time qRT-PCR, data collection, analysis, interpretation and writing of the article. AL-S: *E. histolytica* culture and treatments, obtaining mRNA. JS-S: 2D-DIGE and function analysis. LM: data interpretation, critical writing and revision of the article. GR: quinoxaline synthesis, OM-C: Mass spectrometry analysis. ER-M: project proposal and design, data interpretation, writing of the article. All authors contributed to the article and approved the submitted version.

## Funding

This work was supported by the Secretaría de Investigación y Posgrado, Instituto Politécnico Nacional (SIP-IPN)-Mexico (projects 20200335, 20211108). RGAB received financial support through the scholarship 285467 granted by SEP/CONACYT-Mexico. ER-M, LM and GR received supports from COFAA-IPN, EDI-IPN and SNI-CONACyT. AL-S received a BEIFI-IPN support and CONACYT fellowship (CVU1008704).

## Conflict of Interest

The authors declare that the research was conducted in the absence of any commercial or financial relationships that could be construed as a potential conflict of interest.

## Publisher’s Note

All claims expressed in this article are solely those of the authors and do not necessarily represent those of their affiliated organizations, or those of the publisher, the editors and the reviewers. Any product that may be evaluated in this article, or claim that may be made by its manufacturer, is not guaranteed or endorsed by the publisher.
